# Increasing children’s physical activity through a teaching-assistant led extracurricular intervention: process evaluation of the action 3:30 randomised feasibility trial

**DOI:** 10.1186/s12889-015-1501-3

**Published:** 2015-02-18

**Authors:** Russell Jago, Simon J Sebire, Ben Davies, Lesley Wood, Kathryn Banfield, Mark J Edwards, Jane E Powell, Alan A Montgomery, Janice L Thompson, Kenneth R Fox

**Affiliations:** Centre for Exercise, Nutrition & Health Sciences, School for Policy Studies, University of Bristol, Bristol, UK; Department of Health and Social Sciences, University of the West of England, Bristol, UK; Nottingham Clinical Trials Unit, University of Nottingham, Nottingham, UK; School of Sport, Exercise and Rehabilitation Sciences, University of Birmingham, Birmingham, UK

**Keywords:** Physical activity, Intervention process evaluation, Interview, Focus group, Children

## Abstract

**Background:**

Many children do not engage in recommended levels of physical activity (PA), highlighting the need to find ways to increase children’s PA. Process evaluations play an important role in improving the science of randomised controlled trials. We recently reported the results of the Action 3:30 cluster randomised feasibility trial illustrating higher levels of moderate to vigorous intensity PA among boys but not girls. The aim of this paper is to report the process evaluation results including intervention fidelity, implementation, context and how intervention components and trial design could be improved before proceeding to a definitive RCT.

**Methods:**

Children’s session enjoyment was assessed every two weeks. Reasons for non-attendance were provided by questionnaire at the end of the intervention. Post intervention interviews were held with participating teaching assistants (TAs) and school key contacts (KCs), and focus groups were conducted with children in all 10 intervention schools. Interviews and focus groups examined how recruitment and session attendance might be improved and established which elements of the programme that were and were not well received.

**Results:**

Data indicated good intervention fidelity with TA’s adopting enjoyment-focussed teaching styles and the sessions improving children’s skills and self-esteem. Several positive aspects of implementation were identified, including high session variety, the opportunity to work in teams, the child-led sessions and the engaging leader style. In terms of context there was evidence that TA’s faced difficulties managing challenging behaviour and that further training in this area was needed. TAs and KCs felt that recruitment could be improved by providing taster sessions during PE lessons and clarifying the days that the clubs would run at the point of recruitment. The programme could be improved to enhance interest for girls, by including training for managing disruptive behaviour and making some activities more age-group appropriate.

**Conclusions:**

Action 3.30 showed promise but could be improved by ensuring age appropriate activities, providing more appeal to girls and improving recruitment through taster sessions and early establishment of days of the week it is to be offered on.

**Trial registration:**

ISRCTN58502739.

**Electronic supplementary material:**

The online version of this article (doi:10.1186/s12889-015-1501-3) contains supplementary material, which is available to authorized users.

## Background

Regular physical activity is associated with improved psychological well-being and lower levels of cardio-metabolic risk factors including elevated insulin, lipoproteins and blood pressure among children and adolescents [1]. A number of studies have shown that large proportions of children and adolescents do not engage in the current recommendation of an hour of moderate-to-vigorous intensity physical activity (MVPA) per day [2-4]. Physical activity levels also decline with age, with a recent meta-analysis reporting an average decline of 7% per year during adolescence [5]. The end of primary (elementary) school is therefore an important period in which children’s physical activity behaviours are established. Systematic reviews of physical activity interventions for children have identified that interventions typically yielded small or no improvements in levels of MVPA [6,7]. There is therefore a need to find new, more effective physical activity interventions.

The majority of physical activity interventions for children and adolescents have been delivered at school during the key contact time of the established curriculum (e.g., changes to physical education delivery) [7]. A possible reason for the failure of these interventions is the difficulty of using curriculum time for activities that are not aligned with core educational aspirations [8,9]. The time allocated to PE in the UK curriculum is also limited to around 2 hours per week and therefore there is limited time within which to help less active children to develop the skills and confidence to be active. As such, extra-curricular interventions which harness the infrastructure of the school (buildings, location, key target audience), but do not impinge on educational time may provide a unique opportunity to facilitate physical activity behaviour change among children and young people [8]. Teaching assistants (TA) work in the classroom setting and help teachers to support children with tasks such as reading, writing, and maths in both large groups and on a one–to-one basis, and are a group that could be trained to deliver physical activity sessions for children. A 2009 systematic review of extra-curricular interventions identified 13 papers reporting the results from 11 different studies of which only one had included objective assessments of children’s physical activity [10]. None of the studies were conducted in the United Kingdom [10] and none included Teaching Assistants. A separate 2009 review highlighted the current methodological weakness of evaluations of after-school interventions and highlighted a need for more well-controlled trials [11]. Thus, there is a need to robustly examine the viability and effectiveness of extra-curricular physical activity interventions in UK schools.

Process evaluations are integral elements of complex behaviour change interventions [12] which scrutinise intervention delivery, receipt and implementation [13-15]. A key goal of a process evaluation is to provide insight into whether an intervention “*does*” or “*does not” work*, that is, whether the intervention in itself is ineffective or whether the lack of effect is due to poor intervention implementation [16]. Key features of a process evaluation are the dose delivered, the reach (or number of people who receive the intervention), fidelity (extent to which the intervention was delivered as planned), implementation (how well the programme was implemented) and context which provides critical information on the environment in which the programme was delivered [13]. Process evaluations are also very helpful during the development and piloting of interventions [17-19]. Process evaluations can capture information on how the intervention may work in a trial setting as well as gather feedback from participants, intervention deliverers and key stakeholders on how the intervention content could be improved to maximise the potential for behaviour change. Information that can enhance the running of a trial such as feedback on maximising recruitment, increasing adherence and maximising attendance can also be obtained and used to refine, and improve the trial design prior to conducting a larger, fully powered randomised controlled trial [19].

We have recently reported the results of the Action 3:30 feasibility trial, which was conducted in 20 primary schools (10 intervention and 10 control schools) [20,21] and focussed on increasing the physical activity levels of Year 5 and 6 pupils (9–11 years of age). The 10 intervention schools received the Action 3:30 intervention, which consisted of a 5-day training programme for two TAs per school, who subsequently co-delivered a 40-session after-school physical activity programme (2 × 60 minute sessions per week, for 20 weeks). Up to 30 Year 5/6 children in each school opted to join the study and agreed to attend the Action 3:30 club if their school was randomised to the intervention group. The 40-sessions included adaptations of traditional team sports, small-sided games, and a session in which the children led the activities. Children attending the Action 3:30 clubs received a hand-out after every four sessions (i.e. every 2 weeks) of activities that had been taught during the sessions that they could practice at home.

The strategies that underpinned the delivery were based on the self-determination theory (SDT) principles of building confidence, autonomy and a sense of belonging [22]. Specifically, the sessions were designed to increase the children’s interest in and enjoyment of physical activity and enjoyment was designed to have been facilitated by the TAs adopting a friendly, engaging communication style, in order to boost the children’s skills, confidence, and self-esteem. Due to space constraints, the degree to which the intervention was delivered in a way that was consistent with SDT will be presented in a separate paper and a link to the publication posted on the project website (http://www.bristol.ac.uk/sps/researchprojectpages/action330/).

The feasibility trial analysis suggested that boys in intervention schools obtained 8.6 more minutes of MVPA on weekdays than boys in the control group at the end of the program, but this was not sustained four months after the programme had ended [21]. There was no evidence of an effect on MVPA among girls. In terms of dose delivered, all 40 sessions were delivered in five of the 10 schools, with 3 schools running 39 sessions, one 38 and one 29 [21]. Analyses showed that the mean attendance across the sessions in the 10 intervention schools was 53%, with considerable between-school variability; one school achieved a mean attendance of 86% with all enrolled pupils attending at least half of all sessions, whereas two schools had a mean attendance of 39% and 36% [21]. Thus, the intervention had a reasonable level of reach and a high dose of the intervention was delivered but more work is needed to increase levels of attendance. While these results add to the evidence base for the potential of extra-curricular interventions, they do not provide information on intervention fidelity, implementation, context or why the intervention appeared to be more effective for boys than girls, and how it could be improved to facilitate the desired behaviour change. Thus, the aim of this paper is to report on the results of the process evaluation of the Action 3:30 intervention, with a particular focus on how the intervention was delivered, investigating why the intervention results differed by sex, and ways in which the intervention could be improved before proceeding to a fully-powered trial.

## Methods

The process evaluation included qualitative and quantitative components. The quantitative data were collected during the intervention period and focussed on intervention fidelity and dose. The qualitative data were collected at the end of the 20-week intervention and consisted of interviews with the TAs who delivered the intervention, interviews with school contacts in both intervention and control schools who facilitated the logistics of the study taking place in their school, and focus groups with intervention participants (children) in all intervention schools. In the sections below we provide an overview of these data including participants, data sources and data interpretation. The study was approved by an ethics committee at the University of Bristol, with written informed consent obtained for all adult participants. As the children were in Years 5 or 6 (9–11 years of age) written informed parental consent was obtained.

### Quantitative data

Participants in intervention schools were asked to complete a perceived enjoyment questionnaire every two weeks. The questionnaire consisted of a single item referring to their enjoyment of that day’s session (“Please circle the number that shows how much you enjoyed Action 3:30 today”). Children responded using a 5-point Likert- type scale ranging from 1 (”Not at all”) to 5 (”A lot”). The mean score at each time was computed for each school as well as an overall mean per week across the schools. A multi-level model, using school, pupil, and occasion as the levels, was used to estimate the proportion of variability in the enjoyment ratings given by each pupil on each occasion that was attributable to each level in the model. Results from this model are interpreted as the amount of variation within pupils, between pupils, and between schools.

To understand whether participation in Action 3:30 was accompanied by a reduction in other after-school activities involving physical activity, children in intervention schools were asked on five occasions (weeks 0, 5, 10, 15 and 20) to report in open-response format what they did on each day that week *after-school but before tea-time* (or dinner time) (“Please tell us what organised activities (or clubs) you do at the moment after school but before tea time.” Responses for each day at each of the five time points were subsequently coded into six categories: 1) Organised team sports; 2) Unstructured activities; 3) Structured PA classes; 4) Structured youth clubs; 5) Structured sedentary activities; and 6) Independent sedentary activities. The six activities were subsequently collapsed into three categories (active events, youth clubs, and sedentary events). For each activity type a total was computed for individual participants by summing the number reported each day. Finally, at the end of the intervention period all participants in intervention schools who had missed at least one club session were asked to indicate how much they agreed with 13 reasons for non-attendance (e.g., *When I signed up I thought it would be different*). The content of the thirteen items was informed by conversations held between project staff, school staff and pupils on reasons why children did not attend. Responses were scored from 1 (Not true for me) to 5 (Very true for me). An average rating for each reason was computed. This was subsequently collapsed into three categories by combining responses of ‘Not true for me’ with ‘Not really true for me’, and ‘Often true for me’ with ‘Sometimes true for me’.

### Qualitative data

The two TAs in each of the ten intervention schools were invited to participate in a semi-structured interview and 18 (16 female) of the 20 agreed to take part. Interviews were conducted by a researcher with qualitative research experience but no other involvement in the project. A topic guide was designed to explore the following: views on the training received, general opinions towards the intervention, participant attendance, the perceived impact of the intervention on participants, possible improvements, and the impact of the study on the TAs themselves. The interviewer also pursued any emergent issues that were not on the original interview guides and these topics were then added to all subsequent interviews in an iterative process.

Key contacts (KCs) were the member of school staff in each of the 20 schools who had responsibility for liaising with the research team about the trial (either intervention or control). Typically, KCs were a head of year or the physical education coordinator within the school. All 20 KCs were invited to take part in a semi-structured interviewed and 12 (6 intervention, 6 control) agreed to participate. The KC interview guide covered the following topics: reasons for the school participating in the study, positives and negatives of the study, the perceived impact of the intervention on participants and the TAs involved, attendance, and possible improvements.

A focus group was conducted in each intervention school (n = 10) with six children who were enrolled in the study per focus group. To ensure that data were collected from a range of participants, the focus group participants were purposively sampled to recruit a boy and a girl from each third of attendance per school (i.e. a total of 3 boys and 3 girls per school). An additional boy and girl were randomly selected from each attendance group as reserves in case of any absences on the day of the focus group. The focus group was semi-structured and the guide included the following topics: why children enrolled, activities that were/were not enjoyed, the impact of the intervention on their physical activity and interest in physical activity, and their opinions regarding the Action 3:30 Leaders.

### Qualitative data analysis

Interviews and focus groups were recorded using an encrypted dictaphone and transcribed verbatim. All transcripts were read and re-read by multiple members of the research team and an initial coding frame constructed by four authors (BD, KB, SJS and RJ). Interviews and child focus groups were imported into NVivo (Version 10, QSR, Southport, UK) and coded as individual case nodes. A thematic framework approach was used for data analysis in which the initial coding framework was applied to the three data sources [23]. Researcher triangulation was employed in which themes were identified by one author and verified by two additional authors. Themes were then developed by combining similar codes and were compared between the different sources of data to provide a rich account of the intervention within and between schools and informants. A framework matrix was then created to enable themes to be compared across different sources of data (i.e., children, TAs and key contacts). Reporting of the qualitative data is consistent with RATS guidance.

## Results

### Quantitative findings

**Figure 1:**
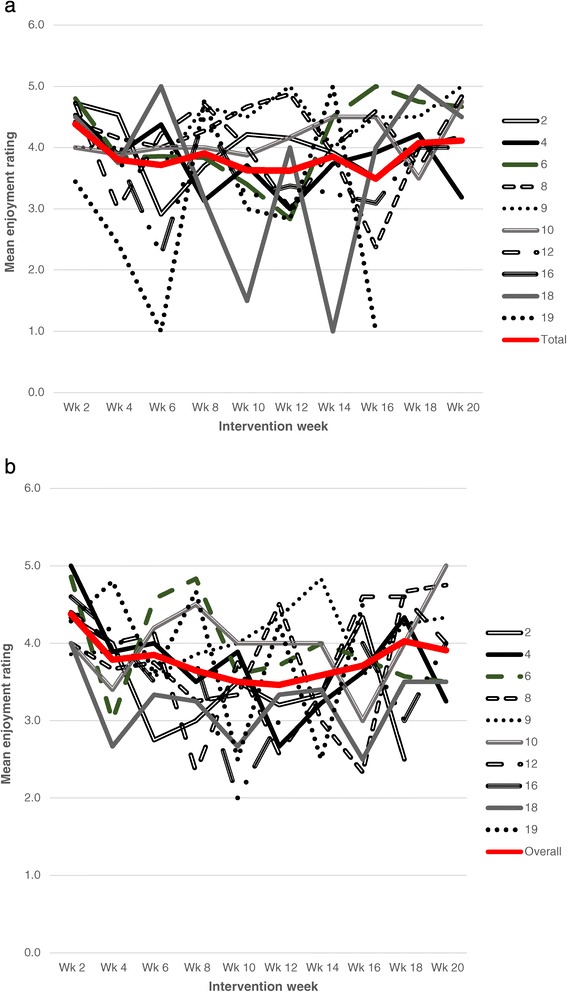
**Mean enjoyment rating (by school and overall) reported by participants over the 20 week Action 3:30 programme. a**: Mean enjoyment rating (by school and overall) reported by participants over the 20 week Action 3:30 programme for girls. **b**: Mean enjoyment rating (by school and overall) reported by participants over the 20 week Action 3:30 programme for boys. Lines represent intervention schools and red line highlights average across schools.

Figures 1a and b shows the mean enjoyment levels per school per session by gender. The data indicate that overall the enjoyment levels were between 3.5 and 4.5, with the majority of sessions between 3.5 and 4.0. The gender-specific graphs indicate similar patterns for boys and girls with a slight downward trend and considerable variability within and between schools. Further, analysis using a multi-level model suggested that less than 1% of the variation in enjoyment scores was between schools, 17% was between pupils, and 82% was within pupils. Thus, most of the variation is unexplained, but probably due to factors such as the content of a particular session and children’s enjoyment differed across sessions.

The mean number of after school activities across all participants for each time point is shown overall and stratified by gender in Table 1. The number of active clubs children engaged in decreased from 3.4 clubs in week 0 to 3.1 in week 20. The number of sedentary events and youth clubs attended each week did not change. Presentation by gender indicated that there were similar patterns for boys and girls.

**Table 1:** **Mean number of active after school activities, youth clubs and sedentary activities reported per week by pupils attending Action 3:30 clubs overall stratified by gender**

	**Active**			Youth clubs*				**Sedentary**	
**All children**									
	N	Mean	SD	N	Mean	SD	N	Mean	SD
Week 0	163	3.4	1.65	48	1.1	0.44	58	1.5	1.01
Week 5	151	3.4	1.72	48	1.1	0.44	61	1.8	1.09
Week 10	134	3.0	1.40	36	1.2	0.59	56	1.7	0.92
Week 15	104	3.3	1.41	34	1.3	0.68	43	1.7	0.97
Week 20	101	3.1	1.50	34	1.1	0.33	51	1.6	0.80
**Girls**									
	N	Mean	SD	N	Mean	SD	N	Mean	SD
Week 0	95	3.4	1.72	31	1.2	0.52	41	1.5	0.75
Week 5	86	3.5	1.76	27	1.1	0.42	39	1.7	0.81
Week 10	85	3.1	1.52	27	1.3	0.66	42	1.8	1.00
Week 15	71	3.3	1.27	24	1.3	0.76	34	1.9	1.02
Week 20	66	3.0	1.34	28	1.1	0.26	34	1.6	0.81
**Boys**									
	N	Mean	SD	N	Mean	SD	N	Mean	SD
Week 0	68	3.5	1.56	17	1.1	0.24	17	1.5	1.51
Week 5	65	3.3	1.67	21	1.1	0.48	22	1.5	1.46
Week 10	49	2.9	1.20	9	1.1	0.33	14	1.5	0.76
Week 15	33	3.2	1.69	10	1.2	0.42	9	1.0	0.00
Week 20	35	3.3	1.70	6	1.3	0.52	16	1.6	0.81

*Any organised youth group including junior Girl Guide/Boy Scout groups (Brownies and Cubs).

**Figure 2:**
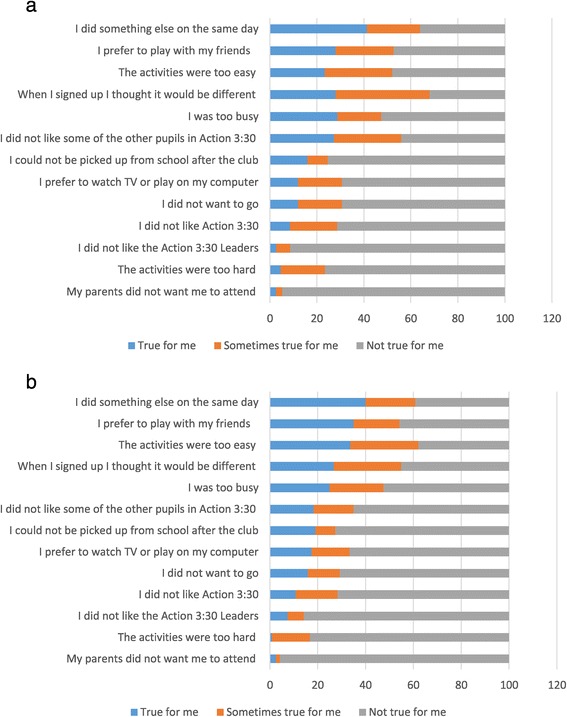
**Summary of pupils’ self-reported reasons for non-attendance at Action 3:30 club sessions. a**: Summary of pupils’ self-reported reasons for non-attendance at Action 3:30 club sessions for girls. **b**: Summary of pupils’ self-reported reasons for non-attendance at Action 3:30 club sessions for boys.

A detailed presentation of the reasons why participants might not have attended the sessions is shown in Additional file 1: Table S1. For the 270 children who provided information, the highest mean value (3.0) was for “*I did something else on the days that Action 3:30 was run*” followed by “*When I signed up I thought it would be different*” and “*I prefer to play with my friends*” which both had mean values of 2.8. These results suggest that logistical issues may have played an important role in limiting attendance at the clubs. It is also noticeable that there were low scores for “*The activities were too hard*” (mean = 1.6), “*I did not like the Action 3:30 leaders*” (mean = 1.4) and “*My parents did not want me to attend*” (mean = 1.2), suggesting that parents supported the programme, session intensity was acceptable and the children liked their TAs. These responses are presented graphically by gender in Figures 2a (girls) and b (boys) but there are no clear gender differences.

### Qualitative findings

In the sections below we have combined the results from the three qualitative data sources to provide an overview of the most important emergent issues and how they relate to the five process evaluation components identified above: 1) recruitment and attendance; 2) fidelity; 3) implementation; 4) context; and 5) key issues to consider in any revision of the intervention.

1. Improving recruitment and attendance

TAs and KCs felt that a taster session in which potential participants have the opportunity to experience an Action 3:30 session would have highlighted the project aims and approach to children and improved recruitment and retention.

*“I think if we could have given a taster beforehand, because I think the children thought it was going to be more of, “You’re going to lose weight. You’re going to do this. You’re going to do that”. I think their perception of what it was, was something slightly different. I think the take-up would have been different had they realized it was such a good after school physical activity club.” (KC, Female, School 6, Intervention).*

*“So we thought perhaps we could’ve, do a taster session for all of our Year 5 s. To come along, have a go, see what they think, because I think, again, people just didn’t know …” (TA, Female, School 6).*

Many TAs felt that recruitment could have been improved by targeting the children that they perceived would benefit most from a physical activity intervention such as overweight or low-active children:

*“I wish we could have chosen the children that attended. I know there was children there that didn’t really get a hundred percent out of it, but children that weren’t allowed to go [not part of the club] would have.” (TA, Female, School 4).*

*“That’s one other thing that could have been maybe changed; was actually involving the TAs beforehand in the actual process of thinking about what kids it would be good to get to do it. You feel like, “Oh, there’s some kids. It would be so good if we could have had that kid join in.” (TA, Male, School 16).*

Teaching assistants and KCs suggested that attendance could have been improved by asking the children to make an agreed commitment to attend the sessions:

*“…I think maybe if there had been a charge or some sort of agreed commitment that made a difference…” (TA, Female, School 19).*

*“…we ask the children to make a commitment… for our children at school is that we usually say that they, they need to do it for at least a term…”(KC, Female, School 4, Intervention).*

A TA suggested that the days the club ran on were selected without knowledge of the children’s commitments. This meant that children couldn’t always attend due to competing engagements:

*“They didn’t know the days. We had to fit it around other clubs here. I was doing a course, so it couldn’t be on Thursdays. We, literally, were forced to do a Monday and a Wednesday, so some of them couldn’t come for those reasons…” (TA, Female, School 2).*

Children also reported that attendance was limited by prior commitments:

*“Well, I couldn’t go any of the Mondays because I was doing another club.” (Participant 6, Male, School 2)*

*“I had a club on one day…Fencing on a Tuesday so we couldn’t come on a Tuesday. So … we came on a Thursday.” (Participant 5, Male, School 16).*

One TA also commented on how low attendance adversely affected the delivery of the sessions:

*[talking about attendance] “It kind of went down. I mean, one time I had five, maybe four people…So it’s very difficult to do the session plan …” (TA, Female, School 9).*

2) Fidelity

Two fidelity-related issues emerged from the interviews: a) the extent to which the TA’s adopted the friendly enjoyment-focussed teaching style in which they were trained; and b) the impact of the intervention on the children’s skill level, confidence, and self-esteem.

- Leader style

In terms of teaching style the children said that their leaders were friendly, enthusiastic and fun and that they encouraged the children to participate in games.

*“I think the 3.30 leaders are incredible…They’re helpful, they explain things to us if we don’t actually understand, and many other things.” (Participant 6, Female, School 4).*

*“They ask if we don’t understand as well.” (Participant 1, Male, School 10).*

*“They were encouraging us to play, and when we didn’t like a game they were kind of encouraging us to like play it and even if we didn’t like it they were encouraging us to play.” (School 6, Participant 5, male).*

b) Impact on motor skills, confidence, and self-esteem

TAs and some children said that the programme improved the children’s physical movement skills. There was also some evidence that there may have been a greater impact on the children who were least confident in sports.

*“In terms of their confidence, their skills, I would say that that would have definitely improved. Their agility and their physical abilities have improved…” (TA, Female, School 6).*

[Talking about the children’s physical skill improvement]*“A couple of children in particular have, because they’re more willing to have a go; they’re not as bad as they thought they were.” (TA, Female, School 8).*

*“…I’m not a very sporty person… not good at sports, but I’ve improved since I’ve done it.” (Participant 6, Male, School 10).*

The KCs and TAs also stated that the children appeared to have increased their self-esteem, especially in those with less confidence in sports. The children said they feel more confident with sports that they experienced for the first time as part of the programme.

*“…and other children, you know, the other by-products have been … virtually all of them feel, or appear to feel much more confident in themselves, have joined other clubs as well. You know, we’ve got virtually a 100 percent take-up rate on, on extracurricular activities now, across the school…”(KC, Male, School 8, Intervention).*

*“I think definitely for some of the girls and a lot of the more sort of timid boys. I think it was brilliant for them…Development and coordination and just confidence as well for girls. Just being able to get stuck in…Their confidence comes out really well.” (TA, Female, School 12)*

*“It got me more confident about some of the sports that I hadn’t done much of… Because there were some sports that… I know and I’m really quite good at and there were sports that I don’t play much and it helped me get my confidence up on it.” (Participant 2, Male, School 12).*

3) Implementation

There were four themes that emerged from the interviews and focus groups which could be grouped under the broader category of implementation. These themes were: a) enjoyment, teamwork and session variety; b) child-led sessions; c) engaging girls in the sessions and d) TA participation.

- Enjoyment, teamwork and session variety

In general the children enjoyed the Action 3:30 sessions, and particularly enjoyed the sense of choice that they were provided with and the ability to be part of a team, both of which were greater than that which children and staff were used to:

“*Well, we expected it be like, like ‘do this, do this!’, but they give us choices, and we had the child- led sessions as well, which I didn’t expect*.” (*Participant 3, Male, School 9*).

*“I think it’s like, I think I’m like, it helped me like work as a team, because most of the games, like it needs like a group to play it…” (Participant 5, Female, School 12).*

*“I think the children understand teamwork better, and I think I do too because you don’t have to be a brilliant player at something.” (TA, Female, School 8).*

*“I think that it has further developed; they’ve got very good team working skills already, the children here have got really good relationships, but I’m sure it’s further developed them in a really structured programme.” (KC, Female, School 10, Intervention).*

The children liked the variety of activities and how the activities were adaptations of traditional games, and that these activities were subsequently changed during the sessions:

*“Like if we were playing that, football, you’d … TA1 and TA2 would change the rules like every five minutes, so then there was this one time when we were playing football and then we were allowed to use our hands” (Participant 5, Male, School 10).*

*“…there’s tons of sports that I like and I just like the way that you play them with the different types of ways and all different rules.” (Participant 3, Male, School 19).*

*“I like Action 3.30 because you can try lots of different sports that you’ve never tried before.” (Participant 2, Male, School 4).*

b) Child-led sessions

The children said that the child-led sessions (where children were encouraged to develop and lead the activities in the session) were often the most enjoyed session.

*So like when the children sessions were on, I really enjoyed it because like we could choose our game.” (Participant 6, Male, School 2).*

*“Because we were allowed to choose what we wanted to do instead of having to follow what Miss chose for us to do.*” (*Participant 3, Female, School 4)*.

*“Because you get to make up your own little discoveries and stuff. I don’t know how to pronounce it better.” (Participant 6, Female, School 4).*

In support of the children’s views, the TAs believed that the child-led sessions helped engage the children and give them a sense of belonging and involvement.

*“I think the way it gets everyone involved worked really well and the fact that the children can be involved in planning the sessions because some of them are pupil-led, so they get involved” (TA, Female, School 19).*

c) Engaging girls

It appears that girls may have found some activities boring and they seemed to have shown less engagement in the club, compared to the boys who sometimes dominated the team games.

*“The children go, ‘Oh, that was boring,’ or, ‘Oh, I don’t want to do that’…especially the girls.”(TA, Female, School 8).*

*“…some of the kids were, some of the girls in particular. It eludes me why they signed up in the first place … they weren’t really that interested.”(TA, Female, School 12).*

*“…the boys just pass to each other and not to the girls, because they don’t really think that they’ll score or anything, and they’ll just let it go to the other team. They just play with each other. It’s not really fun because all the girls just stand around, waiting for someone to … the ball to come to them, but it never does so they don’t really get to play.” (Participant 2, Female, School 10).*

*“Yeah, and that the boys would never pass to the girls.” (Participant 3, Female, School 16).*

d) TA participation and involvement in the sessions

The children enjoyed it when the TAs joined in with the activities and this appeared to be popular when implemented.

*“…our leaders…they don’t mind and they join in sometimes. It just makes it quite enjoyable.” (Participant 3, Male, School 6).*

*“I found that they joined in with the games, which made us feel a lot more good with the games, because we knew they were not just sitting around watching us do it.” (Participant 6, Female, School 9).*

4) Context

In terms of context, the key issue that emerged was the TA’s ability to manage challenging behaviour. There was a clear sense from both the children and the TAs that it was hard for the TAs to manage challenging behaviour. TAs suggested that the training could incorporate more behaviour management training.

*“Maybe there wasn’t quite enough [training] in terms of how to…what scenarios to do when you’ve got children that are difficult, to what to do. If there’s persistent problems, what’s the … what should you do with these kind of children?”(TA, Male, School 16).*

*“[talking about behaviour management training] That probably would have been more beneficial than … I know we needed lots of practical stuff… we could’ve shared ideas more and I think we did in one, the booster session, but not in any great detail.“(TA, Female, School 2).*

Children also identified the disruption caused by challenging behaviour and that the TAs could have exerted more control over it:

*“It was like, loads of kids shouting and screaming and doing disrupting and stuff. So it was really hard to like, get on with what we’re actually doing with the sport” (Participant 2, Female, School 19)*

*“Sometimes they [TAs] could be not strict enough and when somebody kind of goes off, they … they kind of, um, not really know what to do that much.”(Participant 5, Male, School 16).*

5) Key issues to consider in any revision of the intervention

There were seven themes that were raised by the pupils and TAs that could be used to refine the intervention. These themes were: a) the age appropriateness of the sessions; b) incorporating the opinions of the children when considering the session content; c) the amount of content in the sessions; d) improving the clarity of the manuals; e) increasing the amount of challenge in the activities; f) changing some specific activities; and g) revising the session hand-outs/homework.

- Age appropriateness

Children thought that some sessions may have been too basic for their age group. In particular, Year six pupils (age 10–11) also had conflicting commitments such as SATS (Standardised Attainment tests). TAs suggested the club might be more appropriate for children in year 5 (age 9–10):

*“They weren’t babyish but they were … [the children] were a bit too old for them [the activities].” (Participant 5, Female, School 8).*

*“…we felt for year 5 and 6, a lot of the games weren’t age appropriate. It's difficult with year 5 and 6 because sometimes you can get a really mature group … a lot of them were in teams outside school as well.” (TA, Female, School 12).*

*“…maybe to grab them that year earlier sort of year four / five, because while they’re still that little bit more open to suggestion and they’re not as worried to look a bit silly in front of their friends, and again the hormones just aren’t there. And they haven’t got the SATs.” (TA, Female, School 8)*

b) Considering the opinions of a range of children:

Although the children believed that they were able to voice their opinion or be involved in decision making, some would have liked the leaders to be more considerate of everyone’s opinions and activity choices, rather than a select few.

*“… rather than listening to everyone or different people, it was the same people. (Participant 1, Male, School 8).*

*“They listened to maybe two or three and like there’s lot in the session, there’s about twenty five in the club so they would only listen to two or three and lots of people were dying to see their rule but they, they just went with the first one they heard usually.” (Participant 2, Female, School 10).*

c) Amount of content per session

Children and TAs suggested that there might be too much content in each session plan within the allocated time. Children would have preferred to spend more time on a smaller range of activities to allow them time to enjoy them:

*”…sometimes we’d have really fun sports that only lasts like, two minutes. And it’s just go along to something boring… I at least want to have time to get into it and start to enjoy it, instead of just doing it for like, two minutes then getting on to something that I don’t like doing*.” *(Participant 3, Male, School 19).*

“*Having it an hour long and trying to fit quite a lot into it, we realized fairly early on that you’re trying to cover too much stuff. The kids were getting a little bit agitated a lot by the changing*.” *(TA, Male, School 16).*

d) Clearer manuals

TAs said that the session plans could be clearer so the activities are easier to understand. One TA suggested using video clips of activities as a resource.

*“There’s things you, maybe some of the games I didn’t quite understand….So, I have to get my head around that and then I would go email [the Action 3:30 trainer]…You know, could you just describe this.” (TA, Female, School 4).*

“*…it would be nice to have a DVD of the different activities and how they are because…in the manual sometimes the games are not explained very clearly sometimes.” (TA, Female, School 19).*

e) More challenging activities

The children said that some activities should be more challenging in order to make them more exciting.

*“You could like add like new rules to make the game…If it’s challenging, then, it be like harder it’d be more fun.” (Participant 5, Male, School 10).*

*“I don’t really find the activities that hard because they were just like, once they were set they didn’t like change anything more harder, they were just not very hard.” (Participant 2, Female, School 6).*

f) Specific activities

Some children said that elimination games (where children could be ‘got out’) were boring.

*“When we done dodgeball first, it was like before we changed it, we had to sit out a little…It’s boring.” (Participant 5, Female, School 9)*

Children wanted activities to reflect the nature of the sports they were based on, and seemed to value these more as “sports that you would really do,” for example regarding a session on athletics and mini-Olympics a child recalled:

*“Because it wasn’t really sports that you would ever have in the Olympics because there was like …we weren’t like, doing sports that you would really do because it was just like jumping over like, these really like, small hurdles.” (Participant 4, Female, School 16).*

g) Hand-outs

The children thought the hand-outs which were provided every four sessions and included activities to play at home (with family and friends) were difficult to use on their own. The hand-outs could have also been more user friendly and included more pictures of the games.

*“I thought most of them would be just for family because it said ‘teach your family and friends.’ So I thought I’d ask my family, but they all said no, so I thought, what was I going do with that on my own?” (Participant 2, Male, School 2).*

*“Like some of the paragraphs of the games to play didn’t have pictures. So, on one of them, I read the information and I liked it, but there was no pictures so I didn’t get the aim of the game, so I was a bit stuck.” (Participant 1, Male, School 10).*

All of the information from the sections, and specifically how it relates to potential revisions of the intervention has been summarised in Tables 2 and 3. Table 2 highlights the general improvements that could be made to the intervention and Table 3 highlights the changes to leader’s manual and training programme.

**Table 2:** **General improvements to the Action 3:30 intervention based on process evaluation findings**

**Source**	**Improvement**	**Reason for improvement**	**Action required**
TA	Identify the commitments of the children before selecting the days the club will run.	Children didn’t know which days the club would run on. This meant some children had other competing commitments and couldn’t always attend.	Identify the best days for the club to run to maximise attendance (i.e., avoiding clashing with alternative school activities)
CFG	Amend hand-outs so they can be used by children on their own in addition to with others (if possible)	The activities on the hand-outs were often designed for use with family and friends but some children said they couldn’t use them when they had no one to play with. The hand-outs could have more pictures of the game being played.	• Adjust games to include more games that can be played solo.
			• Include more pictures of the game being played.
			• Include more games that don’t need special equipment or suggest alternatives
TA & CFG	Revise programme to increase engagement of girls	TAs: Girls were less interested in the club and presented a greater challenge than the boys in terms of motivation. CFG: Boys tended to dominate team games	• Adapt session plans to include activities that will engage girls and identify ways to make session more appealing to girls (i.e. less direct completion and more within person goal setting and monitoring)
TA, CFG & KC	More age appropriate sessions	TAs: Some of the games were too easy (especially for those who played sports outside of school);	• Amend sessions that are too easy
			• Lower age group to year 5
TA & KC	Conduct intervention in Year 5 s not 5 & 6	Year 6 s tend to be less enthusiastic (especially girls) & have SATS, & thus a younger group may have higher attendance.	

CFG = Child focus group; KC = Key contact; TA = Teaching Assistant; SATs = Standard Attainment Tests.

**Table 3:** **Proposed changes to the Action 3:30 leaders’ manual and training programme based on process evaluation findings**

	**Leaders manual**		
**Source**	**Improvement**	**Reason for improvement**	**Action required**
TA, CFG	Reduce the amount of content in each session	TAs: Children would become agitated when having to regularly change activities; CFG: More time should be given to one activity so it can be played properly.	• Amend individual session plans by reducing number of activities and allowing time for children to experience and master tasks as well as making them more difficult
TA	Improve the clarity of the session plans	Some TAs found some sessions hard to understand. A DVD/video of activities could be a helpful resource.	• Review sessions plans for clarity
			• Produce a DVD of activities or series of online videos that TAs can refer to.
CFG	Changes to specific activities	1) Exclusion games (e.g. dodge ball) can be boring. Children wanted activities to reflect the nature of the sports they were based on, and seemed to value these more as “sports that you would really do”	1) Include options for TAs to utilise to keep all children involved in an activity (instead of sitting out)-cover this more in training.
			2) Include athletics activities that the children can relate to & avoid activities such as running laps.
	**Training**		
**Source**	**Improvement**	**Reason for improvement**	**Action required**
TA & CFG	More behaviour management training	TA: Dealing with bad behaviour was disruptive to club but the training didn’t cover enough on behaviour management	• There should be less practical & more time allocated to behaviour management & for TAs to share ideas.
TA	Training could allow time for TAs to share ideas	Instead of mostly practical based training, scheduling time for TAs to share their ideas on running the club would be helpful.	• TAs may want to seek an agreed behaviour policy with school, so they can act confidently.
CFG	Adapt activities to make them more challenging	Games should be made more challenging to keep them exciting to the children (add new rules & twists).	• More training on adapting games to make them more challenging
CFG	TAs should get involved with activities (if possible)	Some children said they liked it when TAs joined in with them; this made the games more enjoyable.	• Encourage TAs to join in with activities as a strategy to aid enjoyment and understanding
CFG	Consider children’s opinions & activity choices & make sure decisions are fair.	Children said that not everyone was listened to when children were given choice over activities or when offering rule changes to games.	• Training should include ways of making sure that everyone gets a fair say in the activities

CFG = Child focus group; KC = Key contact; TA = Teaching Assistant.

## Discussion

The data presented in this paper have shown that the Action 3:30 intervention was implemented reasonably well but that some revisions to the content which consider the context in which it is delivered are necessary before further evaluation is warranted. Specifically, the children, KCs and TAs liked many elements of the Action 3:30 clubs. Specific elements of the intervention that were well-received included enjoyment of the sessions, the opportunity to build teamwork, the range of choice across all activities (particularly the child-led sessions), and the engaging, empowering TA leadership style. These positive elements of the sessions were perceived to have led to increases in the motor skill-level, the confidence and self-esteem of many of the children, and particularly children who were not previously active. Thus, the data presented in this paper have shown that it is possible to train TAs to deliver enjoyable and effective physical activity programmes after school.

The exit questionnaire data showed that there was a small reduction in attendance at other types of clubs among the intervention group over the period that the programme was running, and that a key reason why the children did not attend Action 3:30 clubs was that they had another after-school commitment. These findings suggest that to some extent the children who attend extra-curricular physical activity programmes will switch from one programme to another, and as a result any intervention effect that might be obtained from new interventions such as Action 3:30 is likely to be attenuated. There was also some evidence that the clubs were more difficult to lead when there were lower levels of attendance, suggesting that attendance might have adversely affected programme delivery. However, we previously reported that the boys who attended the Action 3:30 clubs were obtaining 8.6 more minutes of MVPA per day than the control group at the end of the intervention period. Thus, it is possible to use extra-curricular provision to increase activity; however, the programmes have to be superior to existing provision. Our findings are therefore consistent with previous research that has identified extra-curricular interventions as a relatively under-explored approach to increasing children’s physical activity [10,20]. However, revisions to the Action 3:30 programme are needed to maximise the potential benefit for all children, including children who may be switching from another after-school programme.

The focus group data showed that the children particularly appreciated having choice and a sense of ownership over the activities they were taught during the sessions. The quantitative data also showed children enjoyed the sessions although it is important to note that the enjoyment ratings were only completed by children attending the sessions and it seems likely that children who did not enjoy the programme would be less likely to attend. Less than 1% of the variation in enjoyment ratings was attributable to between-school differences suggesting that sessions were delivered consistently in different schools, and session leaders were comparable across schools. The Action 3:30 intervention therefore holds promise as an intervention approach.

Recruitment could have been improved by allowing potential participants to attend taster sessions, perhaps during physical education lessons as part of the recruitment process. This finding is consistent with our recent experience in which we have found that running dance taster sessions is an important route to successful recruitment of girls into an extra-curricular dance intervention [24-27]. Moreover, attendance is likely to have been enhanced by identifying at the point of recruitment, the days of the week when potential sessions would run, and agreeing attendance commitments with the children. Each of these three ideas would be consistent with standard processes for extra-curricular physical activity programmes in schools, and therefore use of these approaches would enhance the external validity of the programme. Implementing these processes into the scope of a trial is, however, more difficult. To facilitate randomisation after baseline data have been collected, it is usually necessary to conduct the recruitment process several months prior to the intervention beginning. Thus, to facilitate the research design, schools need to agree to hold time slots in their extra-curricular timetable, identify potential staff to attend sessions and to facilitate a fair test of the intervention by specifically not offering an alternative programme if their school is assigned to the control arm. All of these options are possible but they require considerable buy-in to the research process that would need to be handled sensitively within the school setting.

The three key stakeholders (participants, TAs and KCs) provided suggestions for general improvements to the Action 3:30 clubs and these are summarised in Table 2. The most significant suggestions were to increase the motivation and interest of girls, and to lower the age group to Years 4 and 5 as opposed to Years 5 and 6. The age appropriateness of the programme was highlighted in both the qualitative findings and the reasons children gave for non-attendance. The mean value for the item “the activities were too easy” was relatively high thereby suggesting that age appropriateness affected both attendance and perhaps also enjoyment and participation. The suggestion to change the programme content to increase its appeal to girls is consistent with the previously published data from this project which has shown that the intervention led to an 8.6 minute increase in the PA of the boys but no effect on PA of the girls. A body of work has shown that boys are generally more active than girls and that there is a steeper decline in the activity levels of girls as compared to boys as they approach adolescence [28-30]. A number of studies have specifically focussed on increasing the activity levels of girls [26,31] but consistent with most child-focussed interventions, the effect of these studies has been limited [6,7]. Thus, it is not clear whether sex-specific programmes or combined programmes are likely to be of greatest benefit. Conversations between the project team and local school staff suggest that it would be logistically challenging to provide separate programmes for boys and girls in UK primary schools. Identifying ways to develop extra-curricular physical activity programmes that appeal to both boys and girls is a challenge that needs to be met. Thus, we feel that the critical revisions that are required are to improve the appeal of sessions to girls and change the focus to Year 4 and 5 pupils.

The three key stakeholders suggested that the session content could be improved by reducing the number of activities per session, using videos to model session plans and finding ways to engage the children more in the activities (Table 3). There were also suggestions that the training would benefit from a renewed focus on how to manage disruptive behaviour and how to adapt the individual sessions to the needs of each particular group. This finding is consistent with our previous work in developing an after-school dance intervention for secondary school aged girls, in which we found that behaviour management is a critical issue, as disruptive students can make delivery of the sessions hard, and negatively affect the atmosphere within the sessions [26,27]. The children also suggested that the programme could be improved by engaging the children themselves in the behaviour management process, setting up agreed conduct codes and ground rules and encouraging the TAs to join in with the sessions. These are all changes that could be incorporated into a revision of the Action 3:30 programme

The findings presented in this paper show that the intervention holds promise and that several key components of the intervention such as session enjoyment, choice over the activities, leadership style and developing the skill and self-esteem of participants may well function as mediators of the intervention in a larger, cluster randomised controlled trial [13,32]. (As noted above, these issues will be addressed in a separate paper which focuses on the SDT aspects of the study). The data also suggest that results may differ for boys and girls. Including assessments of each of these constructs in a future trial would therefore be helpful in understanding intervention effectiveness. The use of process evaluations to inform revisions to complex interventions is consistent with the MRC framework for complex interventions [12]. A number of studies have reported process evaluations of physical activity based cluster randomized controlled trials [33,34] or the use of process evaluations to refine and improve PA interventions through an iterative process [35]. There has, however, been a relative lack of published studies that have reported on the process evaluations of feasibility trials of physical activity interventions [11]. As such, the data presented in this paper provide a unique and valuable example of how a process evaluation can be incorporated into feasibility trials and used to inform the refinement of key intervention components before proceeding to a full trial.

### Strengths and limitations

The major strength of this study is the availability of quantitative and qualitative data from all of the key stakeholders in the intervention. These various sources of information have enabled us to increase our understanding of how the intervention was received and perhaps, more importantly, how it could be improved. The major limitation of the study is that the data reflect the views of stakeholders who received a particular intervention in the Southwest of England, limiting the potential to extrapolate to other extra-curricular interventions in other contexts.

## Conclusion

The Action 3:30 intervention holds considerable promise with the data reported in this paper showing that many children enjoyed the intervention, that the session content was well received by the children and school staff, and that the programme was perceived to have positive effects on the self-esteem and physical activity skill level of many children. The programme could be improved by making the sessions more appealing to girls and focussing on Year 4 and 5 children. In terms of trial design, recruitment into the trial could have been improved by providing taster sessions of Action 3:30 sessions during PE lessons and clarifying the days in which the clubs would run at the point of recruitment.

### Availability of supplementary data

Anonymised versions of the data from the Action 3:30 project have been deposited in the University of Bristol Research Data Repository (http://data.bris.ac.uk/data/) and will be made available to external collaborators from September 2016.

## Additional file

Additional file 1: Table S1. Pupils’ self-reported reasons for non-attendance at Action 3:30 club sessions*.
