# Supplementary material for: Increasing children’s physical activity through a teaching-assistant led extracurricular intervention: process evaluation of the action 3:30 randomised feasibility trial
Source: BMC Public Health. 2015 Feb 18;15:156. doi: 10.1186/s12889-015-1501-3 (PMC4340639; doi:10.1186/s12889-015-1501-3)
Supplement: Additional file 1: Table S1. — Pupils’ self-reported reasons for non-attendance at Action 3:30 club sessions*. [file 12889_2015_1501_MOESM1_ESM.docx]

**Additional file 1: Table S1: Pupils’ self-reported reasons for non-attendance at Action 3:30 club sessions***

|  | **Not true for me** | | **Not really true for me** | | **Sometimes true for me** | | **Often true for me** | | **Very true for me** | | **Overall** | |
| --- | --- | --- | --- | --- | --- | --- | --- | --- | --- | --- | --- | --- |
|  | n | % | n | % | n | % | n | % | n | % | Mean | SD |
| I did something else on the days that Action 3:30 was run | 79 | 29.3 | 22 | 8.1 | 59 | 21.9 | 39 | 14.4 | 71 | 26.3 | 3.0 | 1.6 |
| I prefer to play with my friends | 78 | 28.9 | 48 | 17.8 | 60 | 22.2 | 32 | 11.9 | 52 | 19.3 | 2.8 | 1.5 |
| The activities were too easy | 71 | 26.4 | 46 | 17.1 | 77 | 28.6 | 36 | 13.4 | 39 | 14.5 | 2.7 | 1.4 |
| When I signed up I thought it would be different | 62 | 23 | 40 | 14.8 | 94 | 34.8 | 43 | 15.9 | 31 | 11.5 | 2.8 | 1.3 |
| I was too busy | 101 | 37.4 | 41 | 15.2 | 55 | 20.4 | 26 | 9.6 | 47 | 17.4 | 2.5 | 1.5 |
| I did not like some of the other pupils in Action 3:30 | 103 | 38.1 | 41 | 15.2 | 63 | 23.3 | 21 | 7.8 | 42 | 15.6 | 2.5 | 1.5 |
| I could not be picked up from school at the time Action 3:30 finished | 183 | 67.8 | 17 | 6.3 | 23 | 8.5 | 12 | 4.4 | 35 | 13 | 1.9 | 1.5 |
| I prefer to watch TV or play on my computer | 124 | 45.9 | 60 | 22.2 | 47 | 17.4 | 9 | 3.3 | 30 | 11.1 | 2.1 | 1.3 |
| I did not want to go | 151 | 55.9 | 38 | 14.1 | 44 | 16.3 | 17 | 6.3 | 20 | 7.4 | 2.0 | 1.3 |
| I did not like Action 3:30 | 147 | 54.4 | 46 | 17 | 51 | 18.9 | 14 | 5.2 | 12 | 4.4 | 1.9 | 1.2 |
| I did not like the Action 3:30 Leaders | 208 | 77 | 32 | 11.9 | 17 | 6.3 | 5 | 1.9 | 8 | 3 | 1.4 | 0.9 |
| The activities were too hard | 165 | 61.6 | 48 | 17.9 | 47 | 17.5 | 5 | 1.9 | 3 | 1.1 | 1.6 | 0.9 |
| My parents did not want me to attend | 239 | 88.5 | 18 | 6.7 | 6 | 2.2 | 2 | 0.7 | 5 | 1.9 | 1.2 | 0.7 |

*In descending order based on the combined values of ‘Often true for me’ and ‘Very true for me’
